# COVID-19 pandemic lockdown: An emotional health perspective of Indians on Twitter

**DOI:** 10.1177/0020764020940741

**Published:** 2020-07-07

**Authors:** Dimple Chehal, Parul Gupta, Payal Gulati

**Affiliations:** Department of Computer Engineering, J.C. Bose University of Science and Technology, YMCA, Faridabad, India

**Keywords:** COVID-19, opinion mining, Twitter, lockdown, e-commerce, human behaviour

## Abstract

**Background:**

Novel corona virus (2019-nCoV) has spread in the world since its first human infection in December 2019. India has also witnessed a rising number of infections since March 2020. The Indian government imposed lockdowns in the nation to control the movement of its citizens thereby confining the spread of the virus. Tweeters resorted to usage of social media platform to express their mind.

**Aim:**

Through this article, an attempt has been made to understand the mind-set of Indian people using Python and R statistical software, during the recent lockdown 2.0 (15 April 2020 to 3 May 2020) and lockdown 3.0 (4 May 2020 to 17 May 2020) through their tweets on the social media platform Twitter. Also, opinion on e-commerce during this pandemic has been analysed.

**Method:**

Analysis has been performed using Python and R statistical software. Also, recent articles related to COVID-19 have been considered and reviewed.

**Result:**

Although the country had a positive approach in lockdown 2.0 with only few instances of sadness, disgust and others, the majority of the people had a negative approach in lockdown 3.0.

**Conclusion:**

This analysis can help the health specialists to understand people’s mind-set, the authorities to take further corresponding measures in washing out the virus and the e-commerce stakeholders to adapt to the changing attitudes by adjusting demand and supply plans accordingly.

## Introduction

Novel Coronavirus (2019-nCoV) has made its presence felt across the world since its inception in December 2019 from Wuhan, China. As per the situation report-147 released by World Health Organization (WHO) on 15 June 2020, there were a total of 7,823,289 cases of this virus reported globally out of which 431,541 cases could not survive due to this virus. Of the total cases reported globally, 471,392 cases surfaced in South-East Asia alone with 12,927 deaths. India is the major contributor to this number in South-East Asia with a total of 332,424 (70.52% of South-East Asia cases, 4.25% of the world) reported cases and 9,520 deaths (73.64% of South-East Asia cases, 2.21% of the global cases; COVID-19 Situation Reports, 2020). The first case in India was reported on 30 January 2020 in Kerala (Kamath et al., 2020). The transmission in India has been classified as cluster of cases by WHO in its report. The source of original transmission has been related to seafood wholesale market in China and bats have been considered as the initial host of this deadly virus whereas pigs or pangolins as the intermediate hosts and snakes as this virus’ repository (Naserghandi et al., 2020). The increase in number of infected cases in humans has lead to theories of transmission from animals to humans. The transmission routes chalked out till now include direct contact, respiratory droplets, fomite transmission and faecal–oral transmission. The symptoms of the infection are fever, dry cough, fatigue, evidence of pneumonia, diarrhoea, nausea, dizziness, abdominal pain and so on. Aged patients and patients with history of hypertension, cardiovascular disease and diabetes require severe care if infected with this virus. Treatment till now involves curing the observed symptoms as vaccine for prevention of this virus has not been developed though blood plasma transfusion has been reported as successful in some cases in India, but this is just an exploration than an approved treatment as its efficiency is still under study (Shajan, 2020). Measures taken to combat the spread of this virus by the government of India include imposing of total lockdown for 21 days (25 March 2020 to 14 April 2020) so as to contain the spread of the virus (‘Coronavirus: India Enters “Total Lockdown” after Spike in Cases’, 2020); extending the lockdown (till 3 May 2020 aka lockdown 2.0; ‘Narendra Modi Speech: PM Extends Lockdown, Congress Asks What About Relief For Poor’, 2020); pushing the lockdown further with eased curbs (till 17 May 2020 aka lockdown 3.0) (Lockdown extension in India: Curbs extended for 2 weeks, with some easing, 2020); relaxed lockdown (till 31 May 2020 aka lockdown 4.0) and dividing the nation into green, orange and red zones (lockdown 3.0: Curbs extended for 2 weeks, with some easing, 2020); rapid testing of citizens in containment area (3ContainmentPlanforLargeOutbreaks, 2020); mandatory wearing of masks (‘Masks Are Mandatory For All Now’, 2020) and social distancing among others. Also, the Indian government airlifted students, professionals, and other willing Indians back to their homeland and set up health check-up, quarantine/isolation camps facilities for them, followed by hand-stamping of citizens with travel history for self-quarantine; helped other nations to evacuate their citizens as per its Neighbour first policy; and extended technical services to help other nations set up their testing laboratories. Thermal screenings of the passengers were also done at the airports itself to identify infected Coronavirus patients (Index of /pdf/press_realease_files, 2020). Also, the government continues to trace the contacts of confirmed cases to break the chain of infections, increase community surveillance, share health bulletins on daily basis, grant COVID-19 testing rights to private laboratories, provide access to public–private partnered mobile application Aarogya Setu that is helping the citizens to assess the risk for getting infected with Coronavirus which has recorded 114 million users till 26 May 2020 (Aarogya Setu Mobile App| MyGov.in, 2020) and set up national relief funds to benefit the masses.

India’s ever growing population poses a threat to contain the virus as practicing social distancing becomes a challenge due to the population density. Compared with Italy’s 206, Spain’s 91 and United States’ 36, India has 464 people/km^2^. It has a massive population of 1,380 million as compared with United States’ 330, Italy’s 60 and Spain’s 46 million (Naserghandi et al., 2020). Moreover, hand, cough hygiene and availability of water are also at reportedly low values which can aid the spread of the virus. Furthermore, high rates of existence of patients with hypertension, diabetes, cardiovascular disease and pneumonia can make the matters worse for the nation. The demand and the supply chain has gone for a toss as cases of product hoarding were reported across several parts of India hinted panic buying among citizens (‘Consumer Goods Fly Off the Shelves as Coronavirus Spreads in India’, 2020). Flouting of quarantine norms and fleeing of infected patients from hospitals reflect the thought process of those affected with this virus (‘COVID-19 Suspect in Punjab Escapes from Hospital, Caught’, 2020). The lockdown has affected physical as well mental well being of people adversely (Andrade, 2020). The challenges faced by India government are to manage the lockdown curbs in times of crop harvesting season; to deal with the loss of jobs across retail, restaurant, tourism, manufacturing, transport and other sectors (‘Coronavirus Impact On India’s Retail Sector’, 2020); to revive the economic crisis due to migration of daily wage labourers to their native place and unavailability of workforce to complete the forthcoming workload; to deal with the burden on health care system due to COVID-19; to uplift the underfunded health care and research system; to avoid the possibility of under detection of COVID-19 cases due to shortage in number of Personal Protective Equipment (PPE) kits, doctors, beds, ventilators and medical staff; and to put an end to mass assembling of citizens for religious purpose among others.

## Sentiment analysis of Indians during COVID-19 lockdown

As India is the second most populated country after China, it becomes imperative to analyse the spread and effects of Coronavirus in India (‘India Likely to Surpass China as World’s Most Populous Country in Next 8 Years: UN’, 2019). Micro blogging website, Twitter has been considered to analyse the sentiments of people in India. Although the sentiment of Indians during the first lockdown was majorly positive with very less instances of disgust, anger and sadness (Barkur et al., 2020), This research attempts to gauge their opinion after the initial lockdown was extended (from 15 April 2020 to 3 May 2020 aka lockdown 2.0) and compare them with those in lockdown 3.0 (from 4 May 2020 to 17 May 2020). Also, an attempt to discover latent topics related to e-commerce using Latent Dirichlet Allocation (LDA) topic modelling technique has been done (Chehal et al., 2020) Recording the sentiment from time to time becomes essential for the government to take necessary actions. These actions/events impact the emotional well being of a person. The authorities after knowing citizen’s emotional state can chalk our policies beneficial to them. Also, e-commerce stakeholders can adjust according to their state and regulate products demand and supply.

### Methodology

**Figure 1.**
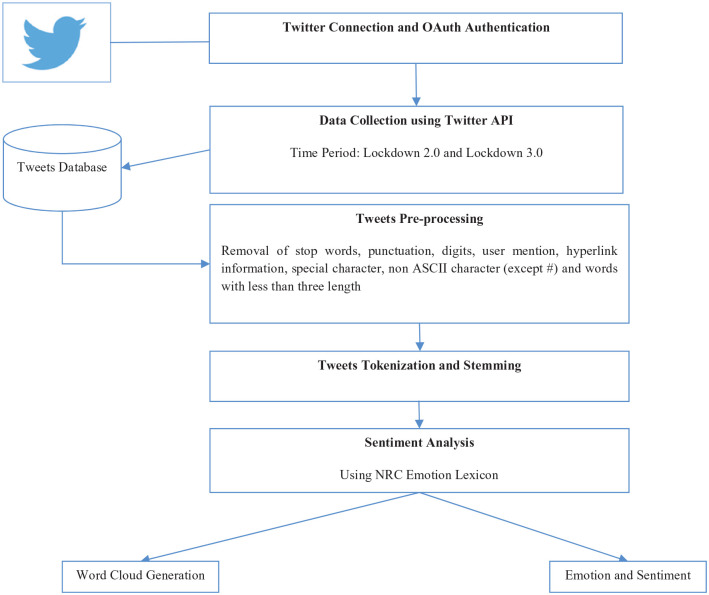
Flowchart of sentiment analysis.

Lockdown 2.0 period was marked with trending of events such as usage of Aarogya Setu app (through #AarogyaSetu); wear your mask challenge (#maskIndia); motivational song by famous Indian personalities (#muskurayegaIndia); mass gatherings for religious purpose (#TablighiJamaat), saluting the Coronavirus warriors of the country – police personnel, doctors, medical and health care staff (#IndiaSalutesCoronaWarriors); and facilitation of special trains for transition of labourers stuck in lockdown to their native places (#ShramikSpecialTrains) to name a few. A total of 29,554 tweets of lockdown 2.0 from social media platform twitter were collected using ‘twitteR’ application programming interface (API) by R. Whereas #IndiaFightsCorona, #LiquorShops, #ShramikSpecialTrains, #MuslimPhobia_In_India, #HumModiKeSathHain, #Say_No_To_Alcohol, #VijaySankalpAgainstCorona and #AatmaNirbharApnaBharat trended during lockdown 3.0. A total of 47,672 tweets were collected for the third lockdown and analysed using ‘syuzhet’ package in R for presence of emotions, namely, positive, negative, trust, fear, joy, anticipation, anger, sadness, surprise and disgust (Jockers, 2016; Figure 1). The process flow to obtain these emotions and sentiment is shown in Figure 1.

After pre-processing the tweets they were analysed using National Research Council Canada (NRC) emotion lexicon to study the feelings of Indians across eight different emotions and two sentiments during this period. This lexicon is a list of English words to which the emotions and sentiments are associated. Thus, each tweet can fall under different emotions and sentiment. As part of pre-processing, stop words, user mentions and hyperlink information was removed from the tweets. Then, all special characters and non-American Standard Code for Information Interchange (ASCII) characters except the hashtag were removed from the refined tweet text. Also, all characters of length less than three alphabets were not considered in tweet analysis (Symeonidis et al., 2018). Finally, tokenization and stemming using Porter Stemmer were carried out to perform the opinion mining.

### Results and discussion

**Figure 2.**
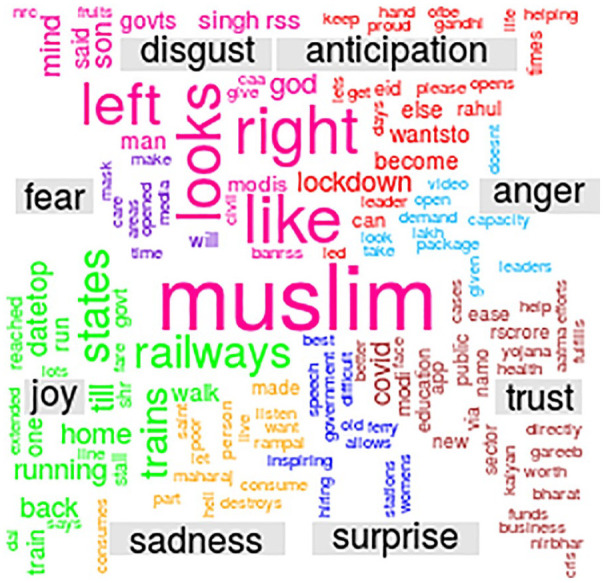
Word cloud analysis of lockdown 3.0 in India.

The analysis was performed using both Python and R language and word cloud depicting the major emotions during lockdown 3.0 was obtained as shown in Figure 2.

**Figure 3.**
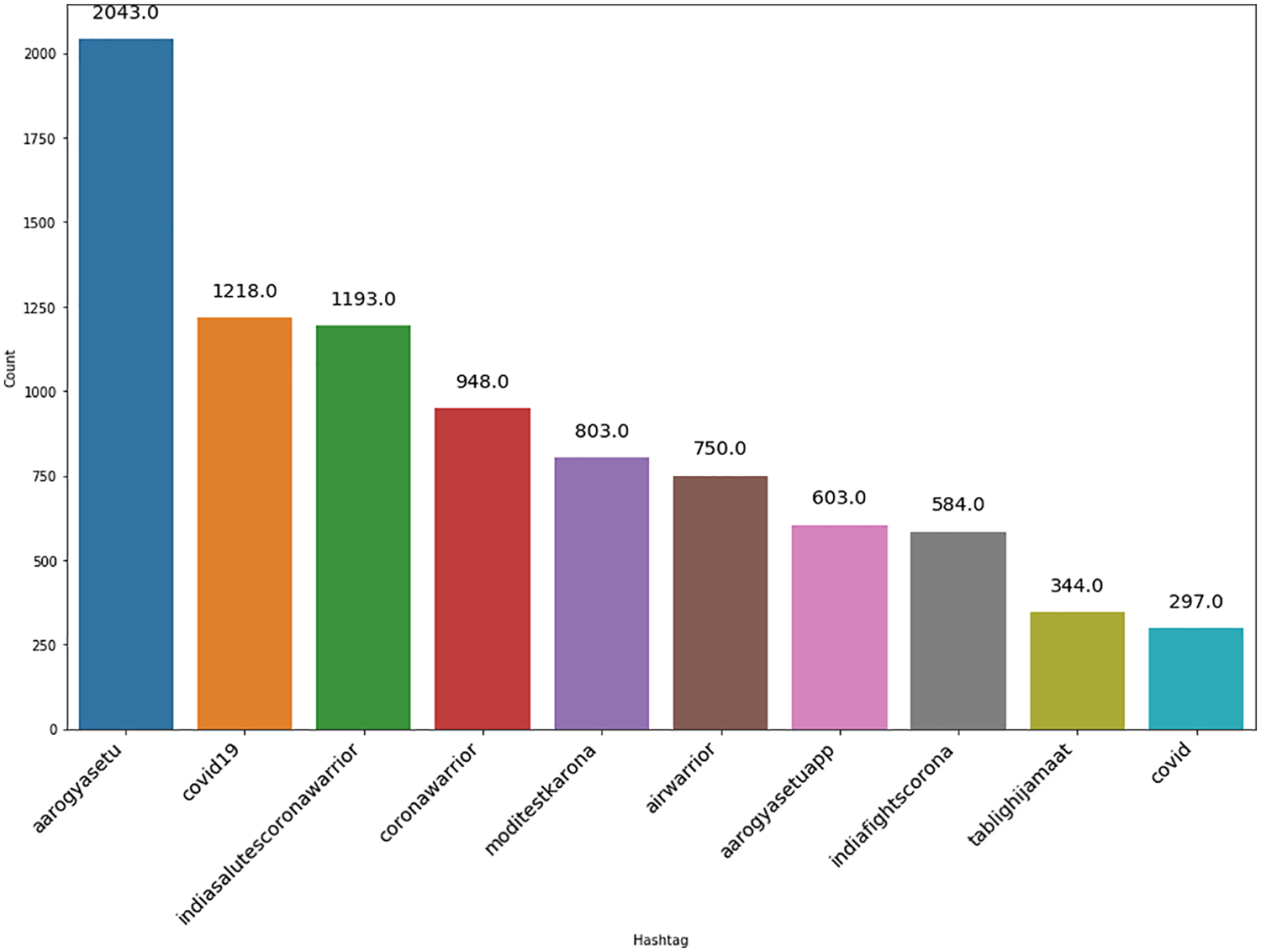
Top 10 positive hashtags during lockdown 2.0 in India.

**Figure 4.**
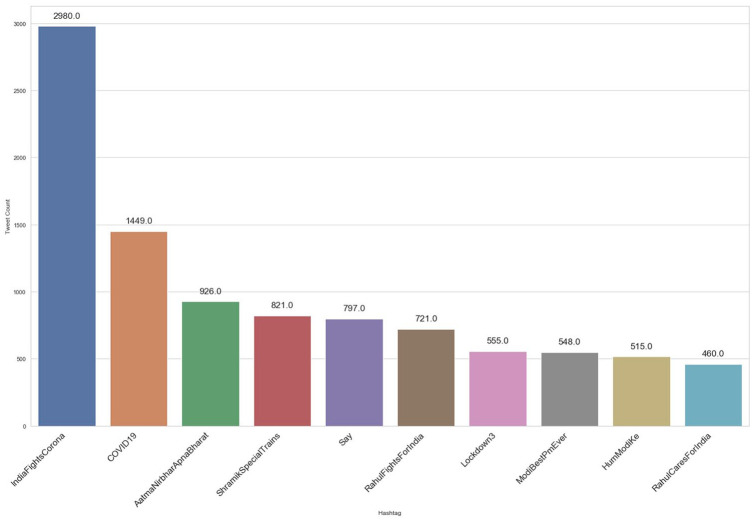
Top 10 positive hashtags during lockdown 3.0 in India.

As shown in Figures 3 and 4, #AarogyaSetu was the top positive hashtag in lockdown 2.0, whereas #IndiaFightsCorona stood out as the top positive hashtag in lockdown 3.0.

**Figure 5.**
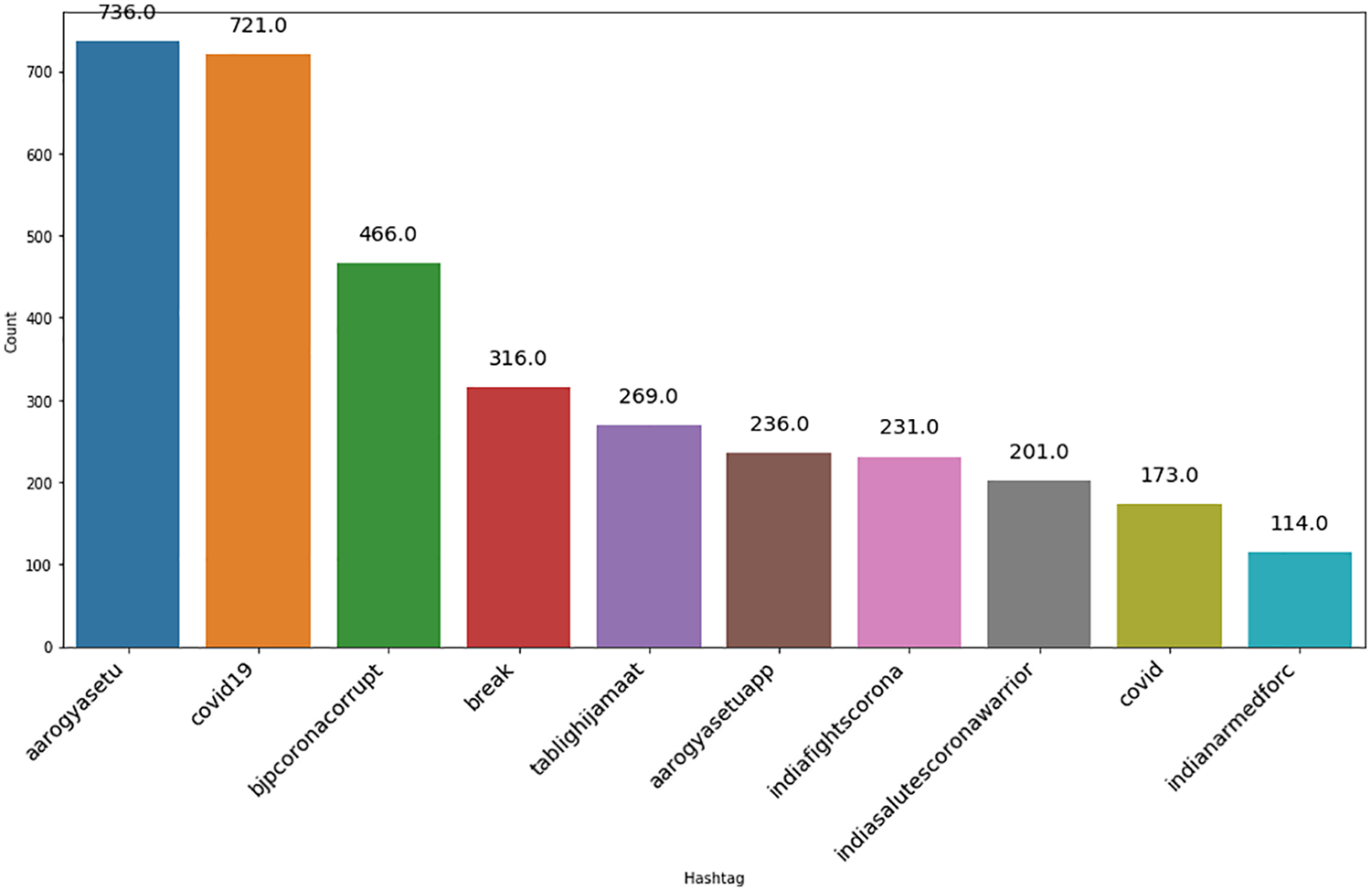
Top 10 negative hashtags during lockdown 2.0 in India.

**Figure 6.**
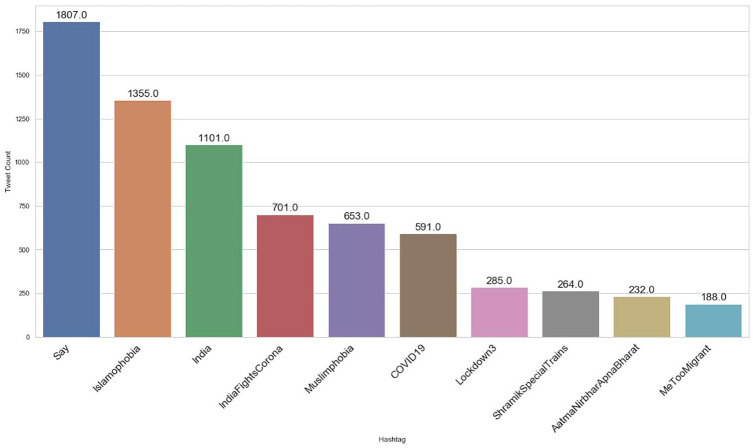
Top 10 negative hashtags during lockdown 3.0 in India.

**Figure 7.**
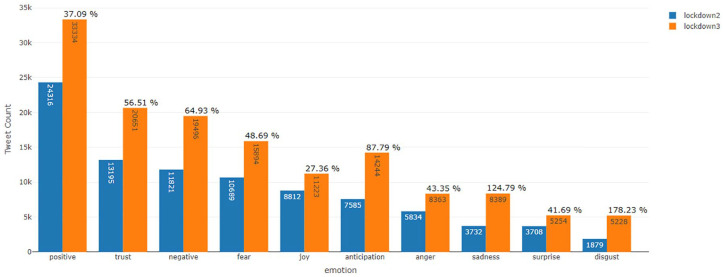
Emotional analysis of twitter users – lockdown 2.0 versus lockdown 3.0.

Also, the top negative hashtag was #covid19 in lockdown 2.0 and #Say_No_To_Alcohol in lockdown 3.0, shown in Figures 5 and 6, respectively. The number of positive tweets for #aarogyasetu, #aarogyasetuapp, #indiafightscorona and #indiasalutescoronawarrior accounted more in number as compared with their negative tweets in lockdown 2.0. Figure 7 represents the comparison of sentiment of twitter users in lockdown 2.0 and lockdown 3.0 for the above mentioned hashtags.

The sentiment of twitter users has been categorized into two sentiments, namely, positive and negative and eight emotions, namely, trust, fear, joy, anticipation, anger, sadness, surprise and disgust. As shown in Table 1, disgust emotion witnessed the highest change in number of tweets (+178.23%) in lockdown 3.0 when compared with its number in lockdown 2.0. The second highest change in number of tweets (+124.79%) across the two lockdowns was witnessed for sadness emotion and the third highest change in number of tweets (+87.79%) was observed for anticipation emotion. This change in number of tweets in lockdown 3.0 from lockdown 2.0, when combined with the results in Figures 6 and 2 suggest it can be due to #Say_No_To_Alcohol and #Islamophobia, the government, the opposition leader, respectively. Table 1 also consists of percentage of an emotion out of total downloaded tweets (29,554 and 47,672 tweets for lockdown 2.0 and 3.0, respectively). While the red coloured downwards facing arrows depict a dip in percentage of a particular emotion in lockdown 3.0 when compared with that in lockdown 2.0, green coloured upwards facing arrows depict a rise in this percentage. The following emotions – anger, fear, joy, surprise, trust and positive sentiment’s percentage ((number of tweets associated to an emotion/total number of downloaded tweets in that lockdown) × 100) in lockdown 3.0 was less than that in lockdown 2.0. But emotions anticipation, disgust, sadness and negative sentiment’s percentage in lockdown 3.0 was more than in lockdown 2.0. It should be noted that the total number of tweets at the bottom of Table 1 is not the numeric total of tweets across the emotions and sentiment but the total number of downloaded tweets for the lockdown period. Also, each tweet can be part of more than one emotion or sentiment due to the usage of NRC emotion lexicon. While social media cannot reflect the sentiment of total population of any country, but it can surely be counted as a sample population to determine the vibe of a nation. The obtained analysis shows that not all went down well in the minds of citizens during the third lockdown. May be this is why the Indian government launched financial stimulus package towards the end of lockdown 3.0 (FM’s COVID stimulus package: Personal finance relief measures, 2020).

**Table 1.** Emotional analysis of twitter users – lockdown 2.0 versus lockdown 3.0.

Emotion	Lockdown 2.0		Lockdown 3.0			Percentage change in no. of tweets from lockdown 2.0 to lockdown 3.0
	Number of tweets	Number of tweets/total tweets downloaded (percentage)	Number of tweets	Number of tweets/total tweets downloaded (percentage)		
Anger	5,834	19.74%	8,363	17.54%	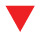	43.35
Anticipation	7,585	25.7%	14,244	29.88%	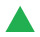	**87.79**
Disgust	1,879	6.4%	5,228	10.97%	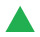	**178.23**
Fear	10,689	36.2%	15,894	33.34%	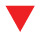	48.69
Joy	8,812	29.8%	11,223	23.54%	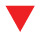	27.36
Negative	11,821	40.0%	19,496	40.90%	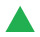	64.93
Positive	24,316	82.3%	33,334	69.92%	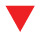	37.09
Sadness	3,732	12.6%	8,389	17.60%	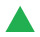	**124.79**
Surprise	3,708	12.5%	5,254	11.02%	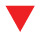	41.69
Trust	13,195	44.6%	20,651	43.32%	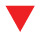	56.51
Total tweets downloaded	**29,554**		**47,672**			

The red coloured downwards facing arrows depict a dip in percentage of a particular emotion in lockdown 3.0 when compared with that in lockdown 2.0, whereas green coloured upwards facing arrows depict a rise in this percentage. The first three highest percentage change in no. of tweets from lockdown 2.0 to lockdown 3.0 are in bold.

## Impact of COVID-19 on e-commerce and consumer shopping behaviour

Understanding the shift in consumer behaviour during epidemics like Coronavirus also becomes vital so that the stakeholders involved can adjust to these changes and act swiftly in response to changing priorities. As per ShipBob’s daily updates of e-commerce sales trends for merchants across verticals, baby products month over month (MoM) sales have surged online, while electronics week over week (WoW) sales have scaled up as on 16 April 2020 (Daily Ecommerce Sales Trends & Resources| ShipBob, 2020). Table 1 lists the MoM and WoW sales across various e-commerce verticals by ShipBob. Amazon reported that its business in India is the most affected due to the Coronavirus lockdown (Abrar, 2020).

As shown in Table 2, while baby products were the clear winner as on 4 May 2020 due to highest MoM sale percentage, it was apparel that stood first due to highest MoM sale percentage as on 21 May 2020. Nutrition WoW sale percentage was the highest as on 21 May 2020 as compared with the rest of the e-commerce categories. This data reflect the consumer behaviour shifting from stocking up of baby products, sports and fitness products to apparel and nutrition, respectively.

**Table 2.** ShipBob’s MoM and WoW e-commerce sales trends.

Vertical	As on 4 May 2020		As on 21 May 2020	
	MoM sale percentage	WoW sale percentage	MoM sale percentage	WoW sale percentage
Baby products	**+693.9**	**–27.1**	**–72.9**	−10.01
Nutrition	−0.2	+8.8	+49.19	**+46.96**
Food and beverage	+12.4	−6.0	+15.9	**–28.62**
Beauty	+64.6	+62.7	+48.73	−8.22
Apparel	+20.4	+62.5	**+95.99**	+6.93
Electronics	+9.4	**+71.7**	+24.94	+2.04
Toys and games	+66.5	+21.9	+44.57	−6.58
Sports and fitness	+112.2	−7.6	+14.99	+1.29
Jewellery	**–39.6**	+0.9	+13.57	+2.33
Household goods	−2.4	+2.8	+72.09	+11.05

MoM: month over month; WoW: week over week.

The bold values represent the highest and lowest sale percentage across various e-commerce verticals.

During Corona times, consumer is worried about the delivery timelines being met by the e-commerce companies (Daily Ecommerce Sales Trends & Resources| ShipBob, 2020), hygiene standards being followed as the last step in delivery reaches the customer’s door and rise in prices of otherwise discounted products sold online once things return to normalcy among other concerns.

Around 1,555 and 1,455 tweets were extracted using twitter API to understand the latent information about e-commerce during lockdown 2.0 and lockdown 3.0, respectively in India. LDA topic modelling technique was implemented on the extracted tweets and number of topics was set to three. As shown in Table 3, in lockdown 2.0, Topic 1 talks about Amazon and flipkart selling items online during lockdown. Topic 2 talks about delivery of processed, packaged and junk food to people during COVID-19. Topic 3 talks about requesting the government to allow Amazon India to deliver goods and products. In lockdown 3.0, Topic 1 talks about delivering of the product- Motorola Edge, non-governmental organization (NGO), AmazonIN and Flipkart. Topic 2 talks about delivery of essential products in lockdown by flipkart. Topic 3 talks about Quiz time mornings with Amazon, a quiz by Amazon for its customers.

**Table 3.** Topics generated for ecommerce tweets using LDA.

Lockdown 2.0						Lockdown 3.0					
Topic #1		Topic #2		Topic #3		Topic #1		Topic #2		Topic #3	
Topic word	*p* value	Topic word	*p* value	Topic word	*p* value	Topic word	*p* value	Topic word	*p* value	Topic word	*p* value
India	.0167	fake	.0412	AmazonIndia	.0395	India	.0315	flipkartsupport	.0248	amazonindia	.0233
AmazonIndia	.0165	deliver	.0412	goods	.0375	FlipkartStories	.0212	order	.019	eligible	.0142
amazon	.015	Paytm	.0408	delivery	.0285	product	.0174	delivery	.0131	QuizTimeMornings WithAmazon	.0133
flipkart	.0146	Walmart	.0401	Allow	.0275	Motorola	.0168	amazon	.0123	Link	.0121
items	.0138	processed	.0394	Government	.0268	deliver	.0163	amazonIN	.0106	quiz	.0117
online	.0099	packaged	.0394	Commerce	.0265	NGOs	.0147	time	.0097	India	.0112
With	.0093	junkfood	.0394	Products	.0254	order	.0136	product	.0097	Offer	.0108
sell	.0088	contributes	.039	Including	.0251	price	.0136	essential	.0091	available	.0092
like	.0084	COVID	.0211	Offers	.0251	amazonIN	.0114	lockdown	.0086	AmazonSpinandWin	.0092
lockdown	.0084	people	.0172	Requests	.0251	Edge	.0109	commerce	.0086	sale	.0092

NGOs: non-governmental organizations.

Here *p* value stands for probability of word in a topic.

## Limitations and conclusion

The tweets which were collected for this study are in English language which might serve as a limitation for the study. Also, the tweet collection had to be done after every week as the free twitter API provided access to tweets from last 7 days only. As the conversion rates have declined and consumer confidence has gone for a fall, it is time for the e-commerce giants to strategise. While some measures have been taken like sale of essential items, sale of non-essential items, there is a lot more to be done to boost this sector’s growth (Coronavirus Ecommerce Data, Impact & Resources: COVID-19, 2020). As the lockdown curbs will be eased in lockdown 4.0, sales are predicted to boost up with delivery restrictions still in place for areas categorized in red zones. The ultimate objective of the ecommerce industry should be uninterrupted and timely availability of indispensable products to prevent panic among customers. This article highlights changing mind-set of people through the lockdowns. A significant number of tweets were witnessed for disgust, sadness and anticipation emotions indicating that the authorities need to buck up. Also, the tweets which were collected for this study were in English language which might serve as a limitation for the study.
